# The importance of the life cycle perspective for safety assessment in the European Commission’s Safe and Sustainable by Design Framework

**DOI:** 10.3389/ftox.2026.1909986

**Published:** 2026-09-04

**Authors:** Irantzu Garmendia Aguirre, Irini Furxhi, Agnieszka Mech, Kirsten Rasmussen, Hubert Rauscher

**Affiliations:** European Commission, Joint Research Centre (JRC), Ispra, Italy

**Keywords:** chemical safety assessment, interdisciplinary collaboration, life cycle perspective/thinking, safe and sustainable by design (SSbD), scoping analysis, value chain collaboration

## Abstract

The European Commission Safe and Sustainable by Design (SSbD) framework represents a paradigm shift in innovation by integrating safety and sustainability considerations from the earliest stages of development, guided by a life cycle perspective. A key driver of this shift is the extension of safety beyond siloed considerations towards a comprehensive life cycle approach. However, this extension of safety to the entire life cycle remains insufficiently understood and is often underestimated or even neglected by practitioners, largely due to a lack of understanding of its practical implications. The life cycle perspective in SSbD is closely linked to another novelty that the framework brings to innovation, the sustainability dimension, which is traditionally addressed through life cycle assessment (LCA) and which inherently captures all life cycle stages of a product for the impact assessment. Likewise, adopting a life cycle perspective in safety requires moving beyond the traditional context-specific safety (substance safety, process safety or product safety). Instead, it requires a more holistic view that considers value chain complexity and safety across all chemicals, processes, products and receptors involved in the SSbD life cycle. This shift requires breaking down long-standing disciplinary silos that were originally developed to manage this value chain complexity. It calls for greater collaboration, building bridges across these disciplines and transparent information sharing across value chains. This paper highlights and explores the importance of the life cycle perspective for safety in the EC SSbD framework. It presents the Scoping Analysis as a central methodological component in the revised framework that strengthens the life cycle thinking in safety.

## Life cycle thinking in safety. The most transformative but misunderstood principle of SSbD

The European Commission’s Safe and Sustainable by Design (SSbD) framework marks a fundamental shift in the governance of chemical and material innovation (Caldeira et al., 2022).

Traditional innovation approaches typically address safety and sustainability only at later innovation stages. By contrast, the EC SSbD framework promotes a cyclic and iterative approach in which both aspects are considered holistically throughout the innovation process from its earliest stages.

A defining feature of the EC SSbD framework is its life cycle perspective, i.e., an approach that considers the safety and sustainability implications of chemicals and materials across their entire life span, from the raw material extraction for their manufacturing, to the end-of-life of the products of which these chemicals and materials are part of (Garmendia I. et al., 2025).

Perhaps the most transformative and challenging aspect of the SSbD framework is its call for a life cycle perspective on safety. While life cycle thinking is well established in sustainability assessment (UNEP/SETAC, 2011), its application to safety represents a more disruptive development in SSbD. Safety assessment has traditionally focused on specific substances [REACH (EU, 2006)], processes [SEVESO (EU, 2012), OSH (EU, 1989), IED (EU, 2024), Waste (EU, 2008)], products (toys, cosmetics, fertilisers …) and associated receptors (workers, consumers, water, air….). In contrast, SSbD requires that safety considerations are extended across all life cycle stages, thereby broadening both the scope and complexity of assessment.

Despite its importance, this expanded perspective is frequently underappreciated in practice, reflecting a limited understanding of its methodological and operational implications, especially in complex value chains.

### Implications of life cycle thinking in safety

Adopting a life cycle perspective in safety requires moving beyond the traditional hazard–exposure–receptor triad logic toward a more holistic view that considers safety across all stages of the SSbD system. This shift requires consideration of a broader range of chemicals and hazards interacting within the system (e.g., co-formulants, additives, auxiliaries …), different uses and varying use conditions and physical and chemical transformations over time that may influence risk.

Consequently, practitioners must define appropriate systems, assessment boundaries, consider indirect and cumulative impacts in the life cycle, and evaluate interactions between safety dimensions that are often addressed separately. For example, a material considered safe during its use (e.g., perovskite solar cells) may pose risks during production or end-of-life treatment (e.g., presence of lead), requiring integrated assessment across these stages.

**FIGURE 1:**
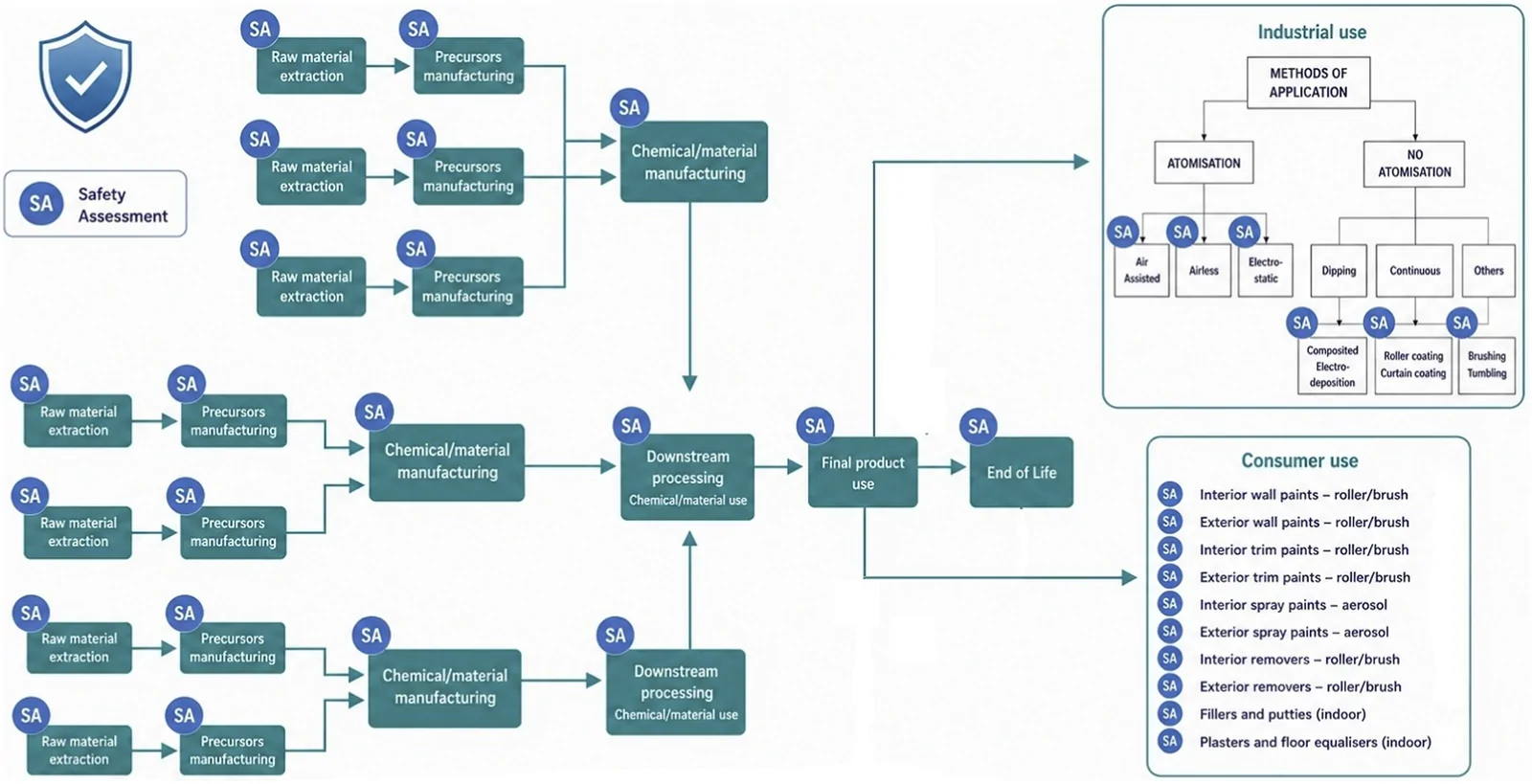
Safety assessment (SA) is needed in every life cycle stage as potential hazards and exposures can occur at every stage. Running a safety assessment in each stage enables risk-based decisions and safer innovation. This example refers to the life cycle of a paint for industrial or consumer use, as indicated in the final product use. Different stages of the life cycle may involve distinct hazards, exposure scenarios, actors and assessment considerations. Adapted from: Garmendia et al., 2026, JRC14686, in press © European Commission. Licence: CC BY 4.0.

Figure 1 illustrates how life cycle thinking in SSbD requires safety assessment at different stages of the chemical/material life cycle. This example refers to the life cycle of a paint for industrial or consumer use, as indicated in the final product use. Different stages of the life cycle may involve distinct chemicals, materials, processes, exposure scenarios, actors, and assessment considerations.

During paint manufacturing, risks may arise from substance handling, mixing operations, or cleaning procedures. For industrial applications, exposure depends strongly on the application method, such as spraying, rolling, dipping, or automated coating, each generating different inhalation or dermal exposure scenarios.

Consumer use may involve different conditions, including lower technical mitigation measures or potential misuse.

Finally, end-of-life processes such as sanding, demolition, incineration, landfill disposal, or recycling may release transformed substances or particles that were not relevant during earlier life-cycle stages. Safety assessment needs to be considered at each of these stages in the value chain. Running a safety assessment at each stage enables risk-based decisions and safer innovation.

This transformation also challenges the traditional compartmentalisation of expertise. Safety assessment has historically been divided across disciplines such as chemistry, toxicology, environmental science, occupational health and safety, and engineering, each typically focusing on specific segments of the life cycle. A life cycle perspective in SSbD requires bridging these domains, with greater attention to methodological alignment, harmonised terminology, and consistency in assumptions and assessment boundaries.

This, in turn, calls for stronger interdisciplinary collaboration and, critically, more transparent information sharing along value chains, including suppliers, manufacturers, users, end-of-life operators and regulators. Such transparency is essential to ensure that relevant data are traceable and accessible across the value chain, enabling more complete and reliable life-cycle-informed safety evaluations.

### Benefits of life cycle thinking in safety

The aim of the European SSbD framework is to guide innovations to develop solutions that are considered safe and sustainable with a life cycle perspective. The framework is a methodology that supports innovation from early stage to market readiness by identifying trade-offs for informed decision-making. The life cycle approach provides the common assessment context for the SSbD and avoids fragmented and incomplete assessment. The life cycle perspective in safety is therefore paramount for a coherent implementation of the SSbD and a consistent and balanced decision-making.

In addition, by extending the safety assessment beyond isolated stages, SSbD ensures that the same substance is evaluated consistently across its uses and transformations, rather than through fragmented, context-specific assessments.

The life cycle approach not only improves assessment consistency but also reduces duplication of efforts and facilitates more coherent use of data across regulatory and sectoral contexts (EC, 2026), in line with the spirit of the One Substance One Assessment (OSOA) approach (EC, 2020). In practice, this means that data generated in one context (e.g., industrial use) can be used and inform assessments in others (e.g., consumer exposure or end-of-life), improving efficiency and consistency while strengthening the scientific basis of decisions.

The life cycle approach encourages value chain engagement. By doing so, it supports early identification of important aspects to be considered along the value chain. It fosters collaboration enabling transparent implementation of SSbD, the identification of “hotspots” along the life cycle, supply-chain traceability and identification of “hotspots” along the life cycle or strategic vulnerabilities linked to geopolitical dependencies and resource security. Identifying such hotspots through value chain collaboration allows practitioners to anticipate risks affecting not only safety but also continuity of supply and industrial resilience. This enables uptake of innovative solutions across the value chain and strengthens value chain collaboration in the long term.

In this context, the EC SSbD Framework (Garmendia I. et al., 2025) acknowledges the need to create trusted environments that facilitate this collaboration, value chain engagement and transparency ambition while preserving business confidentiality and intellectual property rights.

In summary, the life cycle consideration enhances the potential of innovative solutions to be sustainable in the short and long term.

## Sources of conceptual and methodological misunderstandings

Throughout more than 3 years of testing the EC SSbD framework and collection of stakeholder feedback, several conceptual and methodological aspects have been identified as potential sources of the misunderstanding of the life cycle perspective in safety (Garmendia Aguirre et al, 2025) and that have been addressed by the revised framework (Garmendia I. et al., 2025).

Table 1 provides an overview of these sources and how they have been addressed in the revised framework.

**TABLE 1:** Conceptual and methodological misunderstandings addressed by the revised framework.

Features	Sources of misunderstanding	Revised SSbD framework
Goal and scope	Safety assessment is inherently targeted and context-specific (e.g., specific substance). Sustainability assessment integrates impacts across the full life cycle of a product	The scoping analysis serves to define the scope of the SSbD implementation ensuring that both safety and sustainability aspects are addressed within a coherent and consistent scope
Life cycle concept	Differences between substance life cycle addressed in safety assessment and product life cycle in LCA.	The scoping analysis defines the SSbD system for safety and sustainability assessments
Assessment approach	Safety is treated as a set of sequential, confined and separate steps	Safety is consolidated into a single, integrated assessment
Safety in SSbD	Safety is (often) wrongly equated with the toxicity and ecotoxicity impact categories in LCA.	It is explicitly recognised that safety assessment and sustainability assessment fulfil different but complementary functions within SSbD and should not be interpreted as interchangeable
Evaluation approach	A system based on aggregation of results and scoring mechanism	The scoping analysis provides structured methodological guidance for the evaluation stage supporting fit for purpose evaluation, trade-off identification and decision making

### Traditional differences in goals and scope of safety and sustainability

Sustainability and safety assessments have traditionally been developed for different purposes and therefore operate with different methodological logic.

On the one hand, safety assessment is inherently targeted and context-specific, focusing on particular substances, hazardous properties, exposure pathways, populations, use conditions and individual life cycle stages (ECHA, 2026). On the other hand, sustainability assessment, especially through LCA-based approaches, generally adopts a holistic systems perspective that integrates impacts across the full life cycle of a product. The reverse, however, is not the case. LCA does not address specific substances, life cycle stages and/or population in isolation, and safety assessment is not performed holistically, i.e., covering the entire value chain. This asymmetry can lead to an unbalanced operationalisation of SSbD.

To address this misalignment the revised framework (Garmendia I. et al., 2025) introduces the Scoping Analysis as the methodology that contextualises the innovation into the SSbD and defines a common assessment context for both safety and sustainability.

### Life cycle concept

The challenges associated with implementing life cycle thinking in safety assessment stem partly from the novelty of SSbD, which integrates sustainability considerations into innovation domains historically dominated by safety science. Sustainability assessment frameworks, particularly life cycle assessment (LCA), are inherently structured around life cycle stages of a system (ISO, 2006; Rosenbaum et al., 2018). By embedding sustainability, the EC SSbD framework naturally expands the assessment to cover all life cycle stages extending it beyond sustainability to the safety dimension. This represents a significant shift from traditional context-specific safety assessment approaches.

To make this life cycle perspective implicit, the SSbD Framework not only refers to chemicals and materials, but also to processes in which these are involved and products of which they are part, as all life stages play a role in safety and sustainability performance. However, this perspective is not always interpreted in the same way by SSbD practitioners, which may lead to unbalanced operationalisation of the framework.

Further complexity arises because the concept of “life cycle” itself is understood differently across disciplines and assessment frameworks (Guinée et al., 2022). Substance life cycle addressed in safety assessment is chemical-centric and refers to the trajectory of an individual substance across its production, use, transformation, and disposal (OECD, 2017). Material life cycle is typically addressed in environmental and resource assessments and concerns the flow and transformation of materials composed of one or more substances (Allwood et al., 2011). In LCA, product life cycle encompasses the full life span of a product, integrating multiple materials and substances within a defined functional context (ISO, 2006). These different perspectives may result in different system boundaries, priorities, and interpretations within SSbD.

To address this inconsistency the revised framework (Garmendia I. et al., 2025) refers to the SSbD system. The framework recognises that in SSbD substances, mixtures, materials and products are linked in a value chain and that one or more chemical, material and product life cycles might need to be framed within the SSbD system.

### Assessment approach

The original SSbD Framework (Caldeira et al., 2022) introduced a stepwise approach for integrating safety and sustainability into chemicals and materials innovation. While the framework explicitly recognised both dimensions as essential pillars of SSbD, its operational structure unintentionally encouraged an asymmetric interpretation based on the traditional approaches of assessing sustainability and safety. This interpretation reflects the established methodologies underpinning each discipline rather than the intention of the framework itself, which aims at a holistic approach. Consequently, with the traditional interpretation in mind, the life cycle perspective becomes strongly associated with sustainability, while safety often remains confined to context-specific assessment.

To address this conceptual imbalance, the revised SSbD Framework (Garmendia I. et al., 2025) explicitly positions safety and sustainability as two complementary and equally important dimensions. Introducing the concept of the SSbD system in the Scoping Analysis ensures that safety and sustainability assessments are performed within the same clearly defined system boundaries. In this way, the revised framework establishes a common life cycle context for both dimensions, promoting methodological consistency, balanced decision-making and a more coherent implementation of SSbD throughout the innovation process.

### Safety in SSbD and safety in LCA

Additional confusion arises due to the safety-related impacts categories on LCA. The European SSbD Framework (Caldeira et al., 2022) acknowledges that while safety is usually an integral part of sustainability, in the context of SSbD safety goes beyond toxicity and ecotoxicity impact categories and includes aspects such as intrinsic properties of chemical and material, safety of the production and processing, including end-of-life and safety during use and final application. The framework also considers that safety is treated as the first aspect of sustainability, onto which other aspects of sustainability are added.

The inclusion of these impact categories (toxicity, ecotoxicity) in environmental sustainability, on the one hand results in double counting safety aspects in SSbD. On the other hand, although these impact categories address “safety”, they differ fundamentally from safety assessments in terms of objectives, methodologies, and metrics (Hauschild et al., 2008; Fantke et al., 2020). Consequently, they are neither interchangeable nor directly comparable to a safety assessment. Nevertheless, these categories are complementary by addressing different needs.

The revised SSbD Framework (Garmendia I. et al., 2025) addresses this challenge by explicitly recognising that safety assessment and sustainability assessment fulfil different but complementary functions within SSbD and should not be interpreted as interchangeable. Rather than considering safety as a subset of sustainability, the revised framework treats safety and sustainability as two balanced dimensions that are assessed in parallel within the same SSbD system and from a common life cycle perspective. This distinction clarifies that toxicity- and ecotoxicity-related impact categories in LCA contribute to the environmental sustainability assessment, whereas safety assessment addresses a broader range of considerations, including the intrinsic properties of chemicals and materials, occupational and process safety, consumer safety, and end-of-life safety. Although both dimensions may evaluate similar phenomena, such as hazardous substances, they do so for different objectives, using different methodologies, assessment endpoints, and decision criteria.

### Evaluation approach: aggregation and scoring

All mentioned differences also call into question the aggregation, scoring and comparison of results without clear contextualisation because the different dimensions included in SSbD are not commensurable, do not operate at the same system level, and are not necessarily assessed with the same scope, purpose, or methodological approaches.

Instead of aggregating heterogeneous indicators into a single score, the revised framework promotes a transparent interpretation of complementary evidence, allowing safety and sustainability results to be considered together while respecting their different scientific purposes and methodological foundations. The Scoping Analysis, through its three elements (definition of the system, description of the intended innovation, identification and engagement with the actors along the life cycle), contextualises the innovation into the SSbD and provides structured methodological guidance for fit-for-purpose evaluation, trade off identification and decision-making.

## The role of scoping analysis in the SSbD framework

To address these conceptual and methodological challenges, the revised SSbD framework (Garmendia I. et al., 2025) establishes the Scoping Analysis as a core component intended to serve as a decision-structuring and contextualisation step of the innovation within SSbD. Through its three elements it frames what is being assessed (SSbD system), for which purpose (definition of the intended innovation) and under which conditions of uncertainty (life cycle actors’ engagement).

### Defining the goal and scope within the SSbD framework of the innovation

The Scoping Analysis serves to define the scope of the SSbD implementation by identifying relevant substances, materials, processes, products and life-cycle stages that need to be considered in the innovation, ensuring that both safety and sustainability aspects are addressed within a coherent and consistent scope, the SSbD System. This step is essential to establish a shared understanding of the system under evaluation and to avoid fragmented or incomplete assessments.

The Scoping Analysis defines the goal of SSbD implementation by defining objectives and principles of the innovation, identifying (re)design actions and defining indicators, criteria and rules for decision-making in each iteration of an innovation. It also highlights the importance of the engagement with all life cycle actors in the implementation of the SSbD, making the life cycle perspective once again the key underpinning principle of the framework.

### Integrating safety and sustainability into innovation

Moreover, the Scoping Analysis facilitates integration between the two disciplines, safety and sustainability, by extending safety assessment toward a more holistic life cycle perspective while providing sustainability assessment with a more focused and application-specific scope (e.g., process-related sustainability).

**FIGURE 2:**
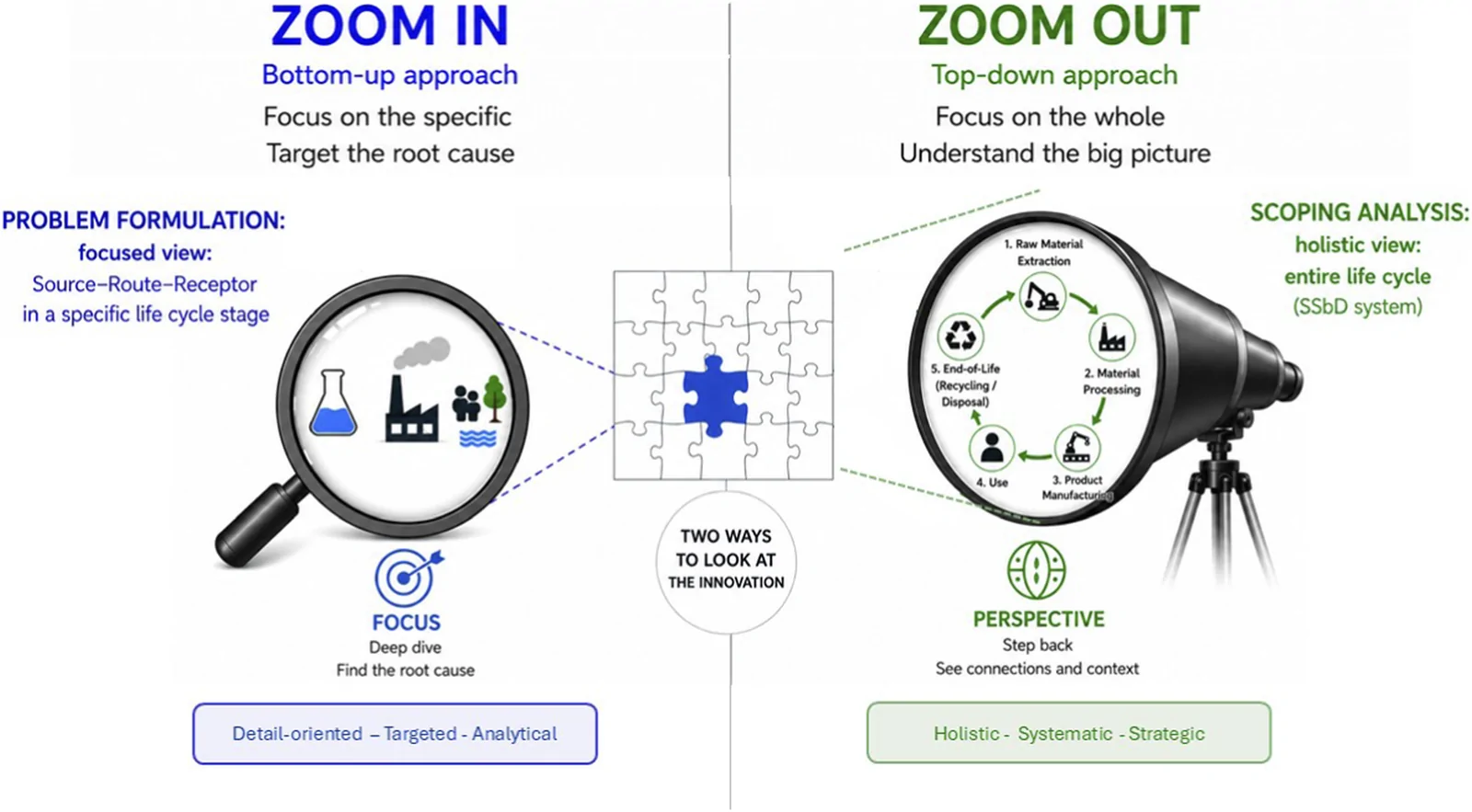
Scoping Analysis (right) and Problem Formulation (left) in SSbD are two complementary ways to look at an innovation. Whereas the former provides a holistic view along the entire life cycle, the latter focuses on source-route-receptor triads in specific life cycle stages. The circular scheme in the right part symbolises the holistic character of the entire SSbD system. Adapted from: Garmendia et al., 2026, JRC14686, in press © European Commission. Licence: CC BY 4.0.

In this way, the Scoping Analysis operationalises the holistic life cycle perspective for safety by explicitly focusing on the completeness of safety considerations within the defined SSbD system with the level of detail adapted to simplified, intermediate, or full safety considerations in the life cycle. At the same time, the Scoping Analysis identifies critical life cycle stages and exposure scenarios that require detailed and robust evaluation in the context of the innovation, ensuring that safety assessments are both comprehensive and proportionate to potential risks (ECHA, 2026; OECD, 2017). While the Scoping Analysis structures and frames the breadth of safety considerations within the SSbD system, the problem formulation delves into the identified critical life cycle stages and exposure scenarios relevant for the innovation and builds on established approaches for targeted and robust assessment grounded in the hazard–exposure–receptor triad, ensuring scientific and methodological rigor. Figure 2 illustrates how Scoping Analysis and Problem Formulation complement each other in SSbD.

### A methodological tool for fit-for-purpose decision-making across innovation stages

Finally, the Scoping Analysis supports the development of SSbD scenarios, which guide iterative assessments and enable coherent decision-making across innovation stages. By defining the system, objectives and decision criteria from the outset, the Scoping Analysis reduces overlaps and inconsistencies, thereby strengthening the robustness and practical applicability of the framework.

## Conclusion: recognising differences while leveraging complementarity

SSbD integrates safety and sustainability in the context of chemicals’ and materials’ life cycle innovation processes. This integration builds on a life cycle perspective that is already widely accepted in the sustainability dimension (UNEP/SETAC, 2011). However, extending this perspective to the safety dimension represents a more profound and challenging transformation.

The incorporation of the Scoping Analysis constitutes a critical advancement in operationalising the SSbD framework (Garmendia et al., 2025a). By defining the SSbD system and identifying relevant life cycle stages, actors, exposure scenarios and safety and sustainability aspects, it supports the integration of targeted and context-specific assessment within a broader life cycle perspective. In this way, safety and sustainability are no longer treated as isolated parallel assessments performed independently but become structurally interconnected across the SSbD system. This enhances clarity, ensures methodological consistency, and enables the integration of safety and sustainability considerations throughout innovation processes.

At the same time, fundamental differences between these disciplines must be acknowledged. Bridging these differences requires improved communication, targeted education, and continued methodological development, particularly at the interface between LCA practitioners and safety scientists.

Ultimately, the effectiveness of the SSbD framework depends on recognising the distinct yet complementary roles of safety and sustainability and embedding both perspectives coherently across the life cycle of chemicals, materials and products. Their appropriate integration, applied at the right stage and for the right purpose, is essential to support informed decision-making and to advance safe and sustainable innovation within the European Union and beyond.

## Data Availability

The original contributions presented in the study are included in the article/supplementary material, further inquiries can be directed to the corresponding authors.
